# Dysregulation of the HOTAIR-miR-152-CAMKIIα Axis in Craniosynostosis Results in Impaired Osteoclast Differentiation

**DOI:** 10.3389/fgene.2022.787734

**Published:** 2022-03-10

**Authors:** Chenbin Dong, Xiangqi Liu, Jun Li, Dongyi Lan, Shan Zheng

**Affiliations:** Department of Plastic Surgery, Children’s Hospital of Fudan University, Shanghai, China

**Keywords:** HOTAIR, miRNA-152, osteoclast, craniosynostosis impact statement, long nocoding RNA

## Abstract

Craniosynostosis is one of the most common craniofacial deformities demanding surgical treatment in infancy. LncRNA HOTAIR has verified its important role in osteogenesis and osteoarthritis. However, whether HOTAIR plays an essential role in the development of craniosynostosis is still unclear. In this study, we aimed to investigate the molecular role of HOTAIR in the osteoclast function and development of craniosynostosis.For osteoclast differentiation, RAW264.7 cells were induced by 50 ng/ml of RANKL and 10 ng/mL M-CSF, followed by TRAP staining. Cell proliferation and apoptosis were assayed by the CCK-8 kit and Annexin V-FITC apoptosis detection kit, respectively. The expression of HOTAIR was determined in PBMCs by qRT-PCR. Protein levels of all those involved genes were measured by Western blot assay. A luciferase reporter assay was used to determine the miRNA target validation. The HOTAIR expression in PBMCs from children with craniosynostosis was significantly downregulated. The results of cell proliferation and apoptosis assays indicated that silencing of HOTAIR could inhibit osteoclast differentiation and increase cell apoptosis. Moreover, the luciferase reporter assay revealed that the regulatory axis and HOTAIR-miR-152-CAMKIIα were the regulatory mechanisms of HOTAIR in the osteoclast function and development of craniosynostosis.In this study, our data showed that HOTAIR could promote osteoclast differentiation by binding miR-152. Furthermore, the HOTAIR/HOTAIR-miR-152-CAMKIIα axis was found to regulate osteoclast differentiation. These results indicate that the HOTAIR plays a crucial role in the development of osteoclasts.

## Introduction

Craniosynostosis is a heterogeneous disease defined as the premature fusion of cranial sutures. It is estimated that one in 2000–2,500 live births are affected (French et al., 1990; Singer et al., 1999; Hukki et al., 2008). This developmental abnormality leads to head strabismus and craniofacial asymmetry in children. It causes permanent neurological, eye, and respiratory dysfunction due to the inability of the cranial crest to adapt to physiological brain growth (Renier et al., 1982; Maliepaard et al., 2014). In recent decades, great progress has been made in technology and safety. Cranial vault reconstruction is still the main method for treating craniosynostosis, with a high incidence of complications and high mortality (Sloan et al., 1997; Clayman et al., 2007; Czerwinski et al., 2010). A comprehensive understanding of the basis of skull sutures is beneficial for the prevention, diagnosis, and treatment of the disease.

A variety of genetic and environmental factors have been reported to be associated with craniosynostosis. Specific single-gene mutations or chromosomal abnormalities account for at least 20% of all cases (Lajeunie et al., 1995; Wilkie, 1997; Singer et al., 1999; Boulet et al., 2008). The identified gene mutations mainly involve upregulation of osteogenesis, downregulation of osteoclast formation, cell proliferation and apoptosis, cell patterning, extracellular matrix regulation, or vascular function (Ma et al., 1996; Muenke et al., 1997; Wilkie, 1997; Fitzpatrick, 2013). Osteoclasts are cells that degrade bone to initiate normal bone remodeling. They mediate bone loss in pathologic conditions by increasing their resorptive activity (Boyce et al., 2009). Besides, skull sutures attain their complex shape at the same age when osteoclast number is highest along concave suture margins (Byron, 2006). Osteogenic dysfunction has been described as a major cause of premature ossification and fusion of the skull (Cohen, 2009). Bone homeostasis depends on a delicate balance between osteoclast and osteoblast-mediated bone resorption and bone deposition, respectively. Osteoclasts are desorbing bone cells derived from macrophage progenitor cells of bone marrow. This plays an essential role in maintaining the stability of the bone’s internal environment.

Osteoclast activation mediated by the receptor activator of nuclear factor-kappa B (RANK)/RANK-ligand (RANKL) signaling plays a critical role in maintaining human cranial suture patency. Dysregulation of osteoclast differentiation and death contribute to premature suture fusion, leading to craniosynostosis (Lyon et al., 2018). Like osteoblast inhibition, osteoclast activation is considered a potential anti-craniosynostosis strategy (Beederman et al., 2014). However, the regulation of osteoclast differentiation in cranial suture biology remains largely unknown.

Long non-coding RNAs (lncRNAs) are a class of non-coding RNA molecules discovered in recent years. They are widely involved in many physiological and pathological processes, such as osteoblast and osteoclast-mediated bone remodeling. HOTAIR is the first trans-acting lncRNA abnormally overexpressed in various tumor tissues and cell lines. Microarray analysis of Xing et al. showed that HOTAIR was expressed in OA cartilage, and its level was higher than that of normal samples (Xing et al., 2014). In addition, it is revealed that the crosstalk between lncRNAs and mRNAs occurs through the competitive binding of microRNAs response elements (Cesana et al., 2011). A recent study showed that HOTAIR could act as a competitive endogenous RNA (ceRNA) for miR-331-3p and miR-124 and regulate their cellular level (Susiarjo et al., 2007). In addition, it is involved in the regulation of the expression of miR-17-5p in osteogenesis. In addition to physical association, HOTAIR could regulate microRNAs levels through direct recognition and target degradation (Huang et al., 2014). Moreover, bone homeostasis depends on the resorption of bones by osteoclasts and the formation of bones by the osteoblasts. Misawa et al., in their study, revealed that HOTAIR inhibits mineralization in osteoblasts. Wei et al. revealed that silencing of HOTAIR promotes osteoblast differentiation by upregulating miR-17-5p expression (Wei et al., 2017; Misawa and Orimo, 2018). However, it remains unclear whether HOTAIR plays a role in osteoclast differentiation.

MicroRNAs (miRNAs) are a class of non-coding RNAs that play essential roles in regulating gene expression. Based on the current state of knowledge, researches on miRNAs in craniosynostosis are scanty. However, Misra et al. stated that transcription factors and microRNA are associated with craniosynostosis (Misra et al., 2019). Through Bioinformatics prediction and RIP analysis, we found that HOTAIR could directly combine with miR-152. Feng et al. found that miR-152 can regulate osteoblast differentiation and influence osteoporosis (Feng et al., 2019). Ma et al. reported that miR-152-3p promotes osteoclastogenesis by targeting osteoclastogenic regulator MAFB (Ma et al., 2021). Moreover, calmodulin is an important regulator of osteoclast differentiation, function, and survival (Seales et al., 2006). It activates multifunctional CAMKs, a serine/threonine-protein kinase family, including CAMKI, CAMKII, and CAMKIV (Soderling and Stull, 2001). It has been reported that the CAMK-CREB pathway regulates osteoclast differentiation and function (Sato et al., 2006). In addition, through the Bioinformatics prediction (TargetScan, PicTar, and miRanda), we determined that Ca2^+^/calmodulin dependent kinases 2-α (CAMKIIα) were the direct target of miR-152. CaMKIIα is an essential mediator of activity-dependent synaptic plasticity that possesses multiple protein functions. It is a critical gate controlling structural, functional, and behavioral expression of synaptic memory. CaMKIIα may result in brain hypoplasia, and craniofacial anomalies included craniosynostosis (Yamagata et al., 2009). Therefore, these data suggest that the HOTAIR-miR-152-CAMKIIα regulatory axis may be involved in osteoclastogenesis during the occurrence and development of cranial suture injury. This study aimed to investigate whether HOTAIR plays a regulatory role in osteoclast differentiation in craniosynostosis development through the miR-152/CAMKII α axis.

## Materials and Methods

### Experiments on Human Study Subjects

The peripheral blood samples of children with craniosynostosis and healthy controls were collected from Children’s Hospital of Fudan University. Peripheral blood mononuclear cells (PBMCs) were obtained from each subject. All patients have signed informed consent. This study has been approved by the research ethics committee of Children’s Hospital of Fudan University (No. 2020-143). The baseline characteristics of the patients are shown in Table 1.

**TABLE 1:** Baseline data of the patients.

No	Gender	Age at operation (month)	Weight at operation (kg)	Craniosynostosis type	Closed cranial suture	Open cranial suture
1	Female	8	8.5	simplex	sagittal suture	Coronal suture on the right
2	Male	16	10	Crouzon syndrome	Bilateral coronal sutures	sagittal suture
3	Male	24	14.5	Apert syndrome	Left temporal squamous suture, left coronal suture	sagittal suture
4	Female	22	11	Apert syndrome	Bilateral coronal suture, right herringbone suture	sagittal suture
5	Female	36	15	simplex	frontal seam	Coronal suture on the right
6	Female	8	9.5	simplex	Coronal suture on the right	sagittal suture
7	Female	9	9	simplex	sagittal suture	Coronal suture on the right
8	Female	21	13	Crouzon syndrome	Whole cranial seam	—
9	Male	47	16.5	Apert syndrome	Whole cranial seam	—
10	Male	6	8	Saethre-Chotzen syndrome	Coronal suture on the right	sagittal suture
11	Male	13	11	simplex	sagittal suture	Coronal suture on the right
12	Female	8	7.5	simplex	Coronal suture on the left	sagittal suture
13	Male	26	12	Crouzon syndrome	Whole cranial seam	—
				Craniosynostosis (n = 13)		Ctrl (n = 26)
male／female, (n)				6/7		13/13
Age, (y)				1.56 ± 0.99		8.38 ± 3.13
weight, (kg)				11.19 ± 2.74		N/A
Syndrome type/simple type, (n)				7/6		N/A

### Cell Culture and Transfection

RAW264.7 cells as osteoclast precursors were obtained from the Cell Bank, Shanghai Institutes for Biological Sciences, Chinese Academy of Sciences. Cells were cultured in Dulbecco’s modified Eagle’s medium (DMEM, Gibco, United States) supplemented with 10% fetal bovine serum (FBS, Gibco, United States). Cells were then incubated at 37°C in a humidified chamber supplemented with 5% CO2. Small interfering RNAs (siRNAs), miR-152 mimics, inhibitors, and negative controls (NC) were purchased from GenePharma (Shanghai, China). They were transfected into cells using Lipofectamine RNAiMAX (Invitrogen), according to the manufacturer’s instructions. The sequences of siRNAs, mimics, and controls are provided as follows: HOTAIR siRNA1, 5′-UAA CAA GAC CAG AGA GCU GTT-3' (sense); HOTAIRsiRNA2, 5′-GCA CAG AGC AAC UCU AUA ATT’ (sense); CAMKIIα, 5′-TTG​TGG​CCC​GGG​AGT​ATT​ACA​GT′-3' (sense); miR-152 mimics, 5′-UCA​GUG​CAU​GAC​AGA​ACU​UGG-3' (sense).

### Osteoclast Differentiation and Tartrate-Resistant Acid Phosphatase (TRAP) Staining

For osteoclast differentiation, RAW264.7 cells (5×10^3^) cells were cultured in the presence of 50 ng/ml of RANKL (#462-TEC; R&D Systems, United States) and 10 ng/mL M-CSF (#416-ML; R&D Systems). Furthermore, for TRAP staining, cells were first washed twice with phosphate-buffered saline (PBS). Then, they were fixed in 4% paraformaldehyde (pH 7.4) at room temperature for 15 min and stained using leukocyte Acid phosphatase Kit 387-A (Sigma-Aldrich, St. Louis, MO, United States) according to the manufacturer’s protocol.

### Cell Apoptosis Assay

According to the manufacturer’s instructions, Annexin V-staining was performed using an Annexin5-FITC apoptosis detection kit (BD Biosciences, United States). Briefly, cells were stained with Annexin5-FITC and propidium iodide in binding buffer for 15 min at 37°C in the dark. The samples were analyzed using a FACScan flow cytometer (BD Biosciences, United States).

### Cell Proliferation Assay

Cells (1×10^3^) were plated in 96-well plates until they reached 30–40% confluence, and 10 μL CCK-8 solutions (Dojindo, Japan) was added to each well. Cells were incubated at 37°C for 2 h. Then, a microplate spectrophotometer (BioTek Synergy HTX Multi-mode Reader, United States) was used to read the OD at 450 nm. Each sample was assessed at 24, 48, 72 h.

### Quantitative Reverse Transcription Polymerase Chain Reaction (qRT-PCR)

According to the manufacturer’s instructions, RNA was extracted from cells using TRIzol Reagent (Invitrogen, United States). cDNA was synthesized using the PrimeScript RT Reagent Kit (Takara, Japan). qRT-PCR was performed using the SYBR Premix Ex Taq RT-PCR kit (Takara, Japan) on LightCycler 480 II (Roche, Switzerland). The expression of miR-152 was quantified using a TaqMan miRNA assay kit (Life Technologies, United States). GAPDH and U6 expression levels were determined as internal controls. Primer sequences for all genes are shown in Table 2.

**TABLE 2:** Primer sequences for all genes.

Primer name	Forward	Reverse
Human GAPDH	5′-GTC​TCC​TCT​GAC​TTC​AAC​AGC​G-3′	5′-ACC​ACC​CTG​TTG​CTG​TAG​CCA​A-3′
Mouse GAPDH	5′-CAG​AAC​ATC​ATC​CCT​GCA​TC-3′	5′-GCA​GAG​CCC​TTT​TTG​ATA​ATG​T-3′
Human HOTAIR	5′-CAG​TGG​GGA​ACT​CTG​ACT​CG-3′	5′-GTGCCTGGTGCT CTCTTACC-3′
Mouse HOTAIR	5′-CAG​TGG​GGA​ACT​CTG​ACT​CG-3′	5′-GTG​CCT​GGT​GCT​CTC​TTA​CC-3′
Mouse NFATc1	5′-GGT​GCC​TTT​TGC​GAG​CAG​TAT​C-3′	5′-CGT​ATG​GAC​CAG​AAT​GTG​ACG​G-3′
Mouse Cathepsin K	5′-AGC​AGA​ACG​GAG​GCA​TTG​ACT​C-3′	5′-CCC​TCT​GCA​TTT​AGC​TGC​CTT​TG-3′
Mouse TRAP	5′-GCG​ACC​ATT​GTT​AGC​CAC​ATA​CG-3′	5′-CGT​TGA​TGT​CGC​ACA​GAG​GGA​T-3′
Mouse RANK	5′-GGA​CAA​CGG​AAT​CAG​ATG​TGG​TC-3′	5′-CCA​CAG​AGA​TGA​AGA​GGA​GCA​G-3′
Mouse CAMKIIα	5′-AGC​CAT​CCT​CAC​CAC​TAT​GCT​G-3′	5′-GTG​TCT​TCG​TCC​TCA​ATG​GTG​G-3′

### Western Blot Assay

The membranes were blocked using 5% BSA in TBST (#ST023; Beyotime, Shanghai, China) at room temperature for 1 h. Western blot assays were performed using antibodies directed against phosphorylated ERK 1/2, ERK 1/2, phosphorylated p65, p65, CAMKIIα (1: 1000; Cell Signaling Technology, United States), and β-Tubulin (1: 5,000; Abcam, UK). Correspondingly, β-Tubulin was used as a loading control. The membrane was incubated with primary antibodies at 4 °C overnight, followed by incubation with HRP-linked anti-rabbit IgG (#7074; Cell Signaling Technology) or anti-mouse IgG (#7076; Cell Signaling Technology) at room temperature for 1 h. The protein bands were visualized using a Pierce ECL Western blotting substrate (#32106; Thermo Fisher Scientific, United States). Images were acquired using a ChemiDoc XRS^+^ System (Bio-Rad, United States).

### RNA Immunoprecipitation Assays

RNA immunoprecipitation (RIP) was performed using the EZ-Magna RIP kit (Millipore, United States). Cell extracts were co-immunoprecipitated with AGO2 (Millipore, United States) or normal rabbit IgG. The retrieved RNA was subjected to RT-qPCR analysis. On the day before transfection, RAW264.7 cells were inoculated in 10 cm Petri dishes with 10^6-10^7 cells in each dish and transfected with miR-152 mimics or an equivalent dose of unrelated control NC. After 48 h, removed culture medium, washed twice with PBS, and scraped cells off with a cell spatula. For each IP test using magnetic separation, 50 μL magnetic beads to 1.5 MLEP tubes, 5 μg IP antibody, RIP wash buffer, proteinase K Buffer, RIP rinse buffer, phenol-chloroform-isoamyl alcohol, chloroform, Salt Solution I 50 μL, Salt Solution II 15 μL, Precipitate Enhancer 5 μL, and Anhydrous alcohol 850 ml were used accordingly. Experiments were carried out following the manufacturer’s instructions. Centrifugation at 4°C, rotation and incubation at 4°C overnight, water bath at 55°C for 30 min, and refrigeration at -80°C were performed. After completing the experiment, the tubes were then air-dried in an ultra-clean table, and the RNA was dissolved in DEPC water. The EP tubes were placed at 60°C for 3 min, and then, real-time PCR was used to detect gene expression.

### Luciferase Reporter Assay

The psiCHECK-2 vector (Promega, Madison, WI) was used to clone the 3′-UTR of CAMKIIα. Primers used were as follows: CAMKIIα 3′-UTR, 5′-CCG​CTC​GAG​TGC​TTC​CCT​CGC​AAA​CT-3' (forward) and 5′- TTA​GCG​GCC​GCT​GGC​TCT​TCC​TCC​CCT​AA-3' (reverse). RAW 264.7 cells were cultured in 96-well, and the cells were rinsed and then harvested 24 h after transfection. The ratio of Renilla luciferase activity to firefly luciferase activity was calculated.

### Statistical Analysis

All statistical analyses were performed using GraphPad Prism 7.0 software. Differences between groups were analyzed using two-tailed Student’s t-test and one-way ANOVA with post-hoc Bonferroni test. *p* < 0.05 was considered statistically significant.

### Animal Experiments

Twelve two-day-old newborn mice were purchased and raised in the SPF animal room for the HOTAIR overexpressed mice model construction and detection. Two c57 female mice were breastfed together. At the age of 5 days, newborn rats were randomly divided into the experimental group (adenovirus loaded with Lnc injected locally) and control group (empty adenovirus/ADV6-NC injected locally into the skull). The animal experiments were approved by the research ethics committee of Children’s Hospital of Fudan University.

### The c57 Newborn Mouse Virus Injection and Sampling

The injection was administered once daily for 4 days (D5 to D8), with a virus titer of 10^9, 20 μL. After disinfecting the operation area with complexed iodine, injected horizontally from the center of the neck, back under the skin using a 10 μL micro-injector. At D9, three mice in each group were sacrificed by cervical dislocation. Their skulls were frozen and stored in liquid nitrogen to extract RNA. The remaining mice in each group continued to be kept for observation. By D20, all mice were sacrificed by cervical dislocation, photographed, and their skulls were fixed in 4% paraformaldehyde for 24 h, and then skull CT was taken. The skull specimen was embedded in paraffin and observed by HE staining. The mice were weighed every 3 days throughout the experiment.

### Total RNA Extraction From C57 Neonatal Rat Skull

The skull was cut into strips, put in a 2 ml flat-bottomed EP tube, added TRIZOL 1ml, and put 2 3.0 mm magnetic beads in each tube. The grinding was carried out in a Mini bead-Beater-16 pearl-Magic Tissue Grinder with 60 Hz power and 2 min duration until no visible bone. After homogenate was obtained, subsequent RNA extraction, reverse transcription, and qRT-PCR were used to detect HOTAIR expression.

### Cranial CT Scan and Data Analysis

After soaking in 4% paraformaldehyde for 24 h, the skull was placed along the long axis in a MICROCT imager (Quantum FX MicroCT). After MicroCT scanning, imported the scanned data into Analyze 12.0 (Mayo Clinical, United States) for data analysis. All the parietal bones were delimited as the target area to obtain bone density and bone mass. According to the standard curve provided by the software, the actual bone mineral density (mg/mm3) = (measured value + 2,769.7)/3.4445.

### Pathological Sections and Staining of the Skull

After completing the CT scan, the skull tissue was removed from paraformaldehyde and immersed in a 10% EDTA decalcification solution. Wax block embedding was completed by the Shanghai Google Biological Co., Ltd. The wax block was taken and cooled in a refrigerator at -20°C for 30 min. Using a slicer, slice the slices to the thickness of 3 μm and bake them in the oven for 30 min. Routine dewaxing, HE staining, dehydration, and seal were performed. Microscopic examination was performed after preparation.

### Statistical Analysis of Data

SPSS 18.0 software was used for statistical analysis, and GraphPad Prism 6.0 software was used for plotting. Continuous variables were compared between the two groups using unpaired Student’s test. The 0.05 difference was considered statistically significant. (*p < 0.05, **p < 0.01, and ***p < 0.001).

## Results

### HOTAIR Promoted Osteoclast Differentiation

#### Knockdown of HOTAIR decreases the RANKL-induced osteoclast differentiation in RAW 264.7 cells

**FIGURE 1:**
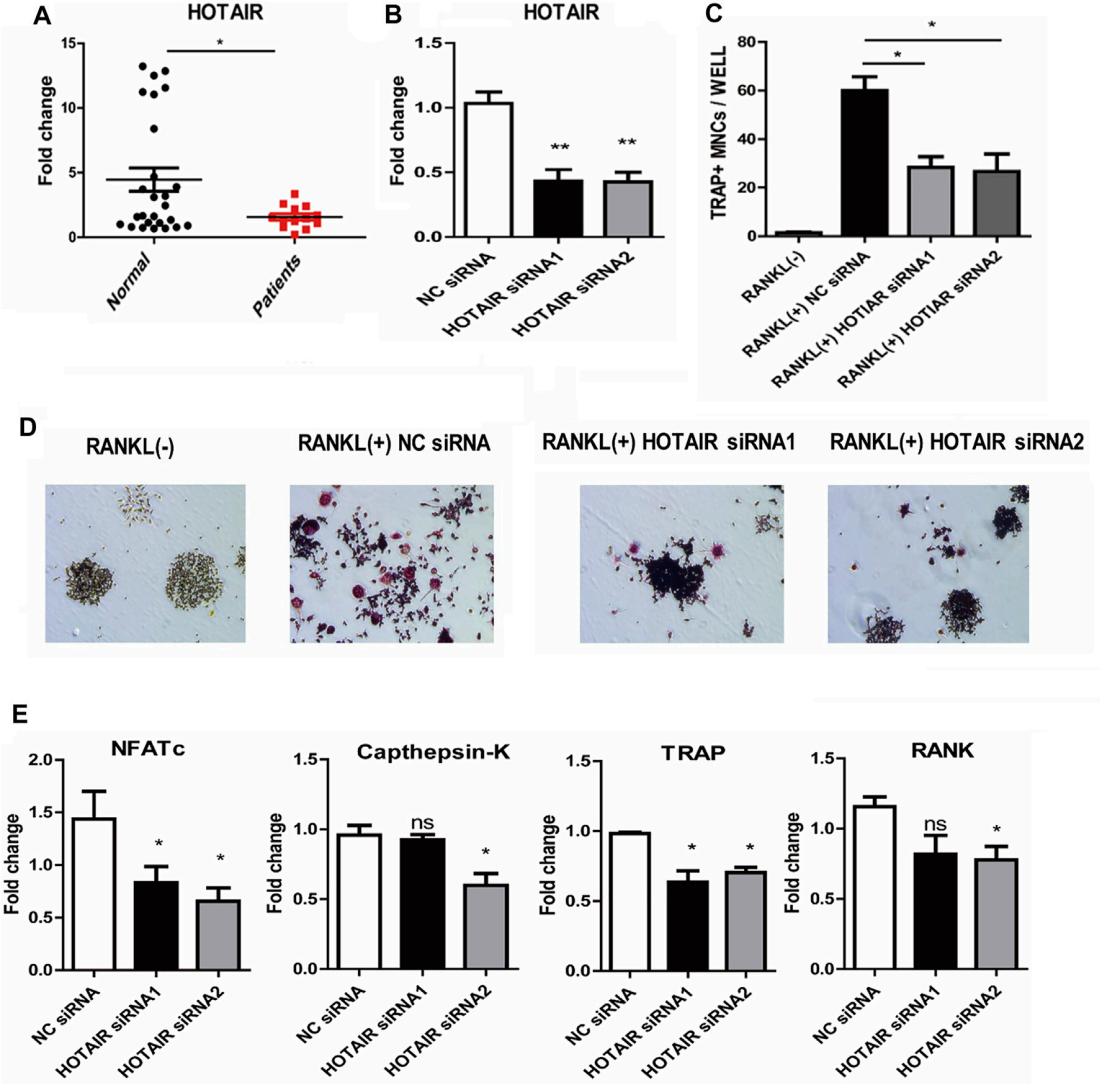
The phenotype of HOTAIR in PBMCs of patients, and knockdown of HOTAIR decreases the RANKL-induced osteoclast differentiation in RAW 264.7 cells **(A)** The levels of HOTAIR were significantly lower in PBMCs of patients with craniosynostosis (N = 13) compared to normal people (N = 26) **(B)** Detection of HOTAIR expression in RAW 264.7 cells with HOTAIR knockdown by two distinct siRNA (200 nM), NC: negative control **(C and D)** RAW 264.7 cells were treated with RANKL and M-CSF for 5 days. Cells were fixed and stained for TRAP. TRAP^+^ cells with more than three nuclei were counted as osteoclasts. Magnification: ×100. TRAP^+^ cells were counted **(E)** The effects of HOTAIR knockdown on mRNA levels of osteoclast differentiation-related genes. Bars represent the mean ± SEM. *p < 0.05, **p < 0.01. The data are representative of three independent experiments.

qRT-PCR results showed that HOTAIR expression was significantly lower in the craniosynostosis children’s group than the control group (*p* < 0.05, Figure 1A). Furthermore, HOTAIR expression was significantly reduced by two specific siRNAs (Figure 1B). RANKL-induced RAW264.7 cells treated with siRNA negative control were able to differentiate into numerous TRAP-positive multinucleated osteoclasts. In contrast, HOTAIR siRNAs treated cells exhibited less TRAP-positive osteoclast formation (Figure 1C,D). Furthermore, the mRNA expression levels of osteoclast differentiation-related genes NFATc1, Cathepsin K, TRAP, and RANK were significantly downregulated in the absence of HOTAIR (Figure 1E).

### HOTAIR Inhibits Apoptosis of Osteoclast Lineage Cells

**FIGURE 2:**
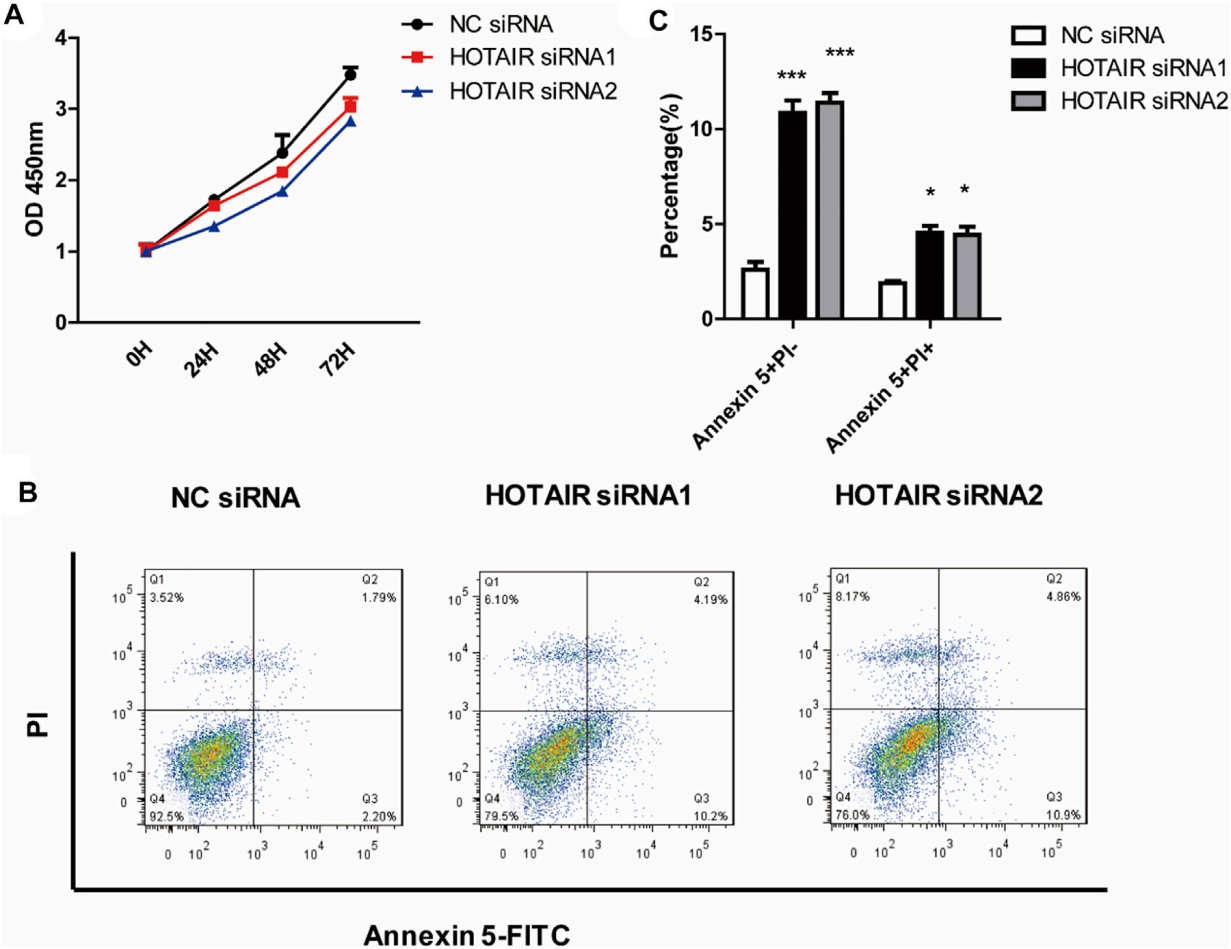
HOTAIR suppresses the apoptosis of mature osteoclasts **(A)** RAW264.7 cells were cultured with M-CSF in the absence or presence of HOTAIR siRNA for 3 days. Then, the proliferation ability of early osteoclast precursors was analyzed by CCK-8 assay **(B)** After transfection with HOTAIR siRNA or the corresponding NCs, RAW 264.7 cells were treated with RANKL and M-CSF for 5 days and stained with Annexin 5-FITC and propidium iodide (PI). Cells were then analyzed using a flow cytometer. Early apoptotic cells were Annexin5^+^ and PI^−^ and late apoptotic cells were Annexin5^+^ and PI^+^ **(C)** Quantification of apoptosis cells was analyzed. Bars represent the mean ± SEM. *p < 0.05, ***p < 0.001. The data are representative of three independent experiments.

The cell proliferation assay results indicated that knockdown of HOTAIR did not affect precursor proliferation during the early process of osteoclast differentiation (Figure 2A). In addition, we evaluated the influence of HOTAIR on the apoptosis of mature osteoclasts. It revealed that silencing of HOTAIR could markedly increase the frequency of both Annexin V single positive, Annexin V, and PI double-positive cell populations (Figure 2B,C). This indicated that more cells undergo apoptosis when lacking HOTAIR expression.

### The HOTAIR-Overexpressing Mouse Model and Phenotype Detection

**FIGURE 3:**
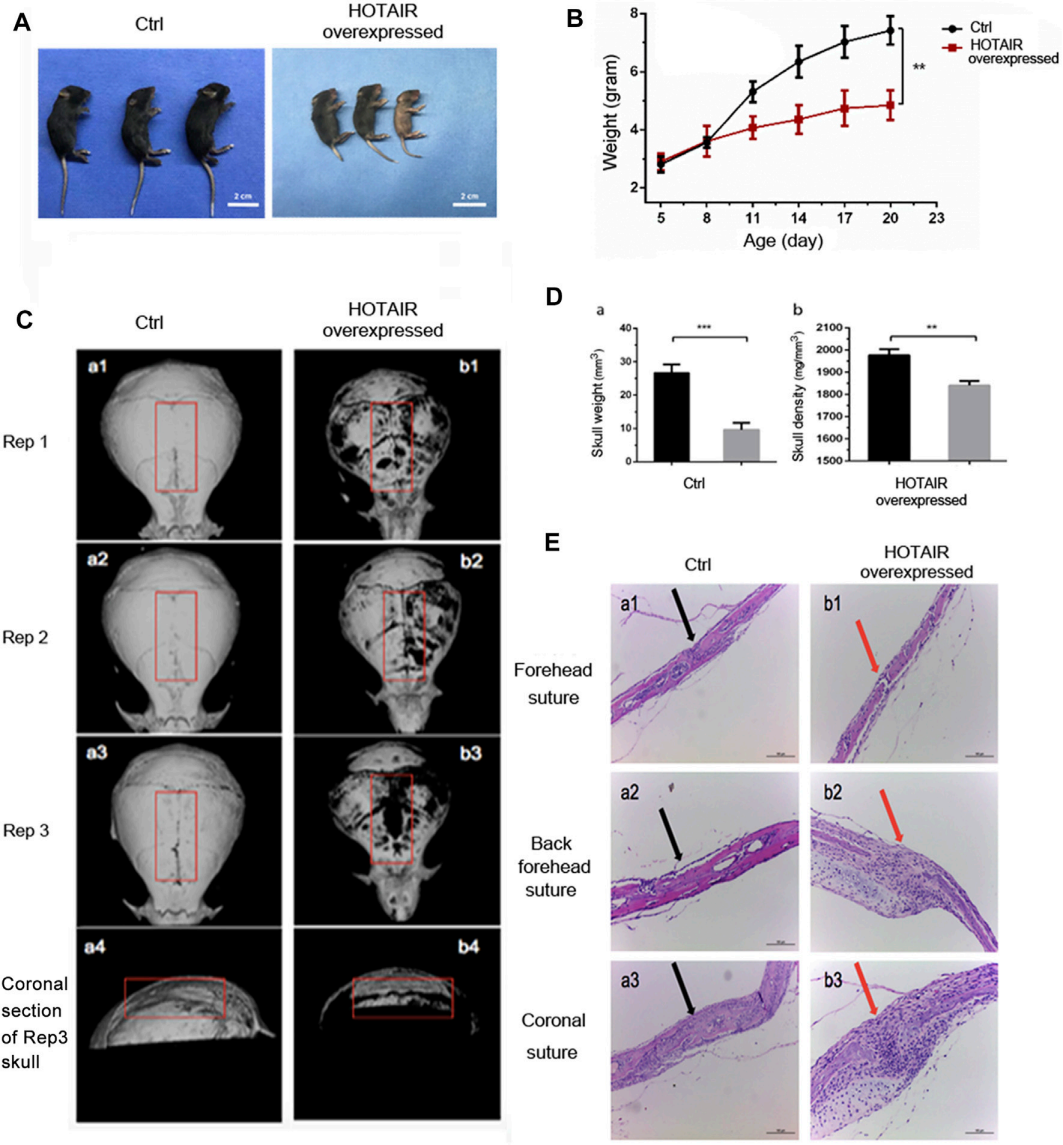
The phenotype of HOTAIR in mouse model **(A)** The effect of HOTAIR on the physical development of C57 mice after 20 days’ birth **(B)** Quantitative assay of the mice weighted along the days after birth **(C)** Images of CT scan of the mouse skull (a1-a3 show the CT scan images of the isolated skulls of three replicates of 20-day-old WT mice, a4 shows the coronal section of a3 skull. b1-b3 shows the CT scan images of the isolated skulls of three replicates of 20-day-old HOTAIR overexpressed mice, b4 show the coronal section of the b3 skull) **(D)** Quantitative assay of the weight and bone density of mouse skull with CT scan (**p < 0.01, ***p < 0.001) **(E)** HE assays of the effect of overexpression of HOTAIR on the skull sutures in mice. Arrows point to the cranial sutures of mice. The scale of the images is 150 um.

Overexpression of HOTAIR was verified by RT-qPCR on D9 post-injection. The relative expression of HOTAIR in the experimental group was 0.0040 ± 0.0012, whereas in the control group was 0 (*p* = 0.041). Also, local injection of HOTAIR loaded with adenovirus was performed, and skull samples were collected 1 day after injection for HOTAIR expression determination. The results showed a significant increase in HOTAIR expression in the skulls of mice in the experimental group. This suggests that local injection of adenovirus-loaded HOTAIR upregulates HOTAIR expression in the skull (*p* < 0.01). In addition, the mice in the experimental group showed a gradual slowdown in growth and development 4 days after injection with short body length, sparse hair, and dark yellow (Figure 3A). Bodyweight has been changed significantly between groups before administration (D5; control group (2.80 ± 0.27 g) vs experimental group (2.90 ± 0.29 g), *p* = 0.68) and at the end of observation (D20; control group (7.41 ± 0.48 g) vs. experimental group (4.84 ± 0.51 g), *p* = 0.0032) (Figure 3B).

Correspondingly, *in vitro* cranial CT scans (Figure 3C) showed that cranial sutures of the experimental group were open and wide, thin skull bones, and bone defects on the surface. On the other hand, skull bone was intact in the control group. The comparison of the coronal section showed that craniosynostosis in the control group was thicker, but narrower in the experimental group (Figure 3C, a4, b4). This suggests that local overexpression of HOTAIR can maintain the cranial suture opening. The cranial CT images were used to delimit the whole cranial range on the Analyze 12.0 software. A significant difference was observed in the average cranial bone mass between the control group (26.67 ± 2.51 mm (Singer et al., 1999)) and the experimental group (9.59 ± 2.08 mm (Singer et al., 1999)) (*p* = 0.0008; n = 3). The mean bone mineral density in the control group (1977 ± 27.06 mg/mm^3^) was significantly higher than that in the experimental group (1841 ± 20.52 mg/mm^3^) (*p* = 0.0022; n = 3). These results demonstrate that skull overexpression of HOTAIR could reduce bone formation, mass, and density (Figure 3D). Moreover, the HE pathological section showed that cranial bones of the posterior frontal suture were overlapped entirely and fused in the control group (Figure 3E, a2), with apparent gaps (Figure 3E). A large number of inflammatory cells could be seen infiltrating between b2 and b3, and the experimental group was thinner that lack obvious plate structure. These indicate that overexpression of HOTAIR could promote osteoclast differentiation, function and maintain cranial suture opening.

HOTAIR positively regulates osteoclast differentiation by downregulating the miR-152 expression.

**FIGURE 4:**
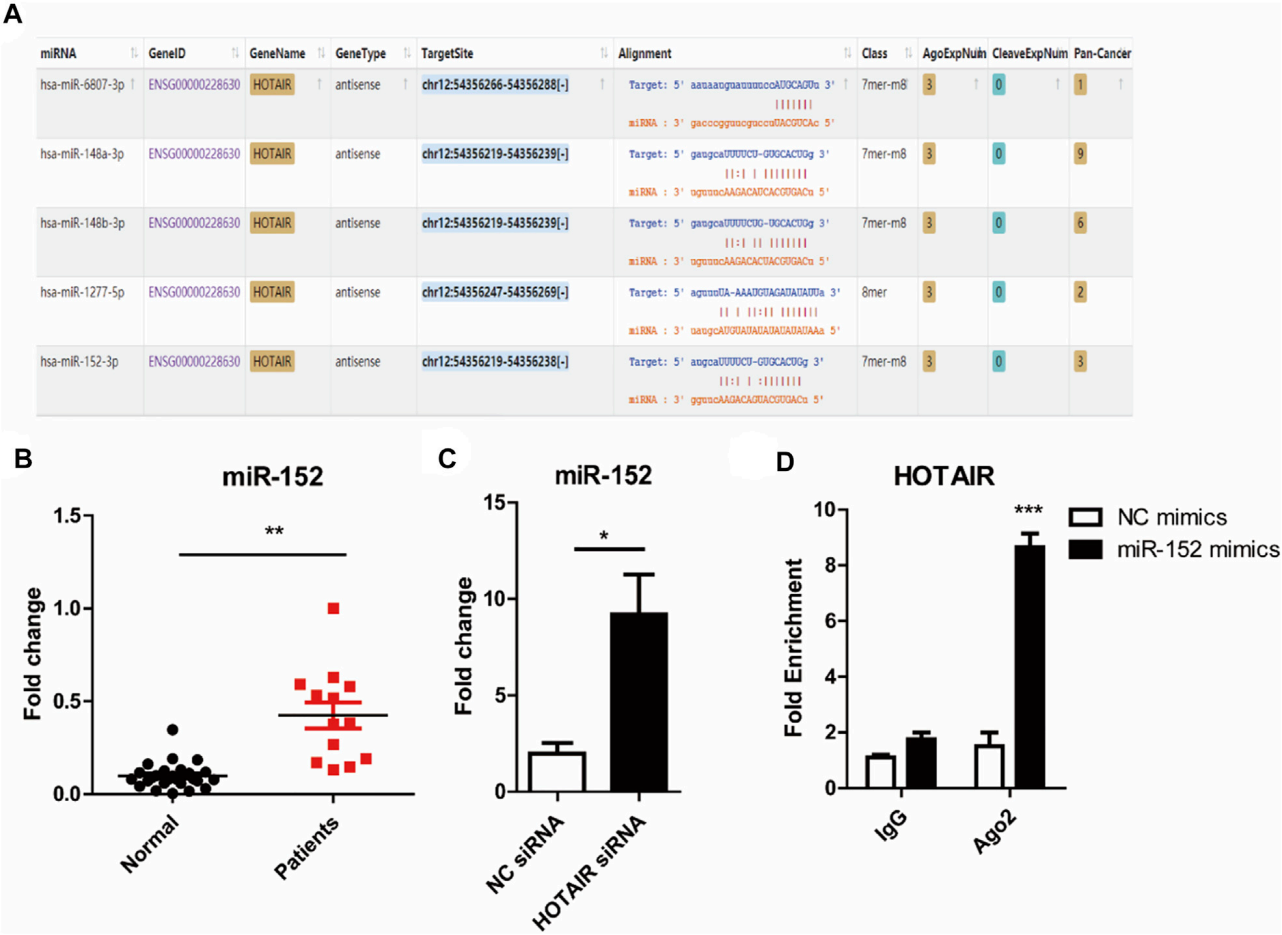
HOTAIR acts as a sponge to inhibit miR-152 expression **(A)** Potential binding domains between HOTAIR transcript and miRNAs through Bioinformatics analysis using starbase Tools. The sequence alignments were visualized following the instruction on https://starbase.sysu.edu.cn/starbase2/index.php **(B)** Detection of miR-152 expression in PBMCs of patients with craniosynostosis, compared to control. *p* < 0.01 (Student’s t-test) were considered statistical significant **(C)** RT-qPCR analysis of miR-152 expression levels in RANKL-induced osteoclasts with HOTAIR knockdown by two distinct shRNAs **(D)** RIP analyses of HOTAIR and miR-152 interaction in RANKL-induced osteoclast with miR-152 mimics or NC transfection (100 nM); RT-qPCR detected the mRNA levels HOTAIR; Bars represent the mean ± SD. *p < 0.05, **p < 0.01, ***p < 0.001. The data are representative of three independent experiments.

**FIGURE 5:**
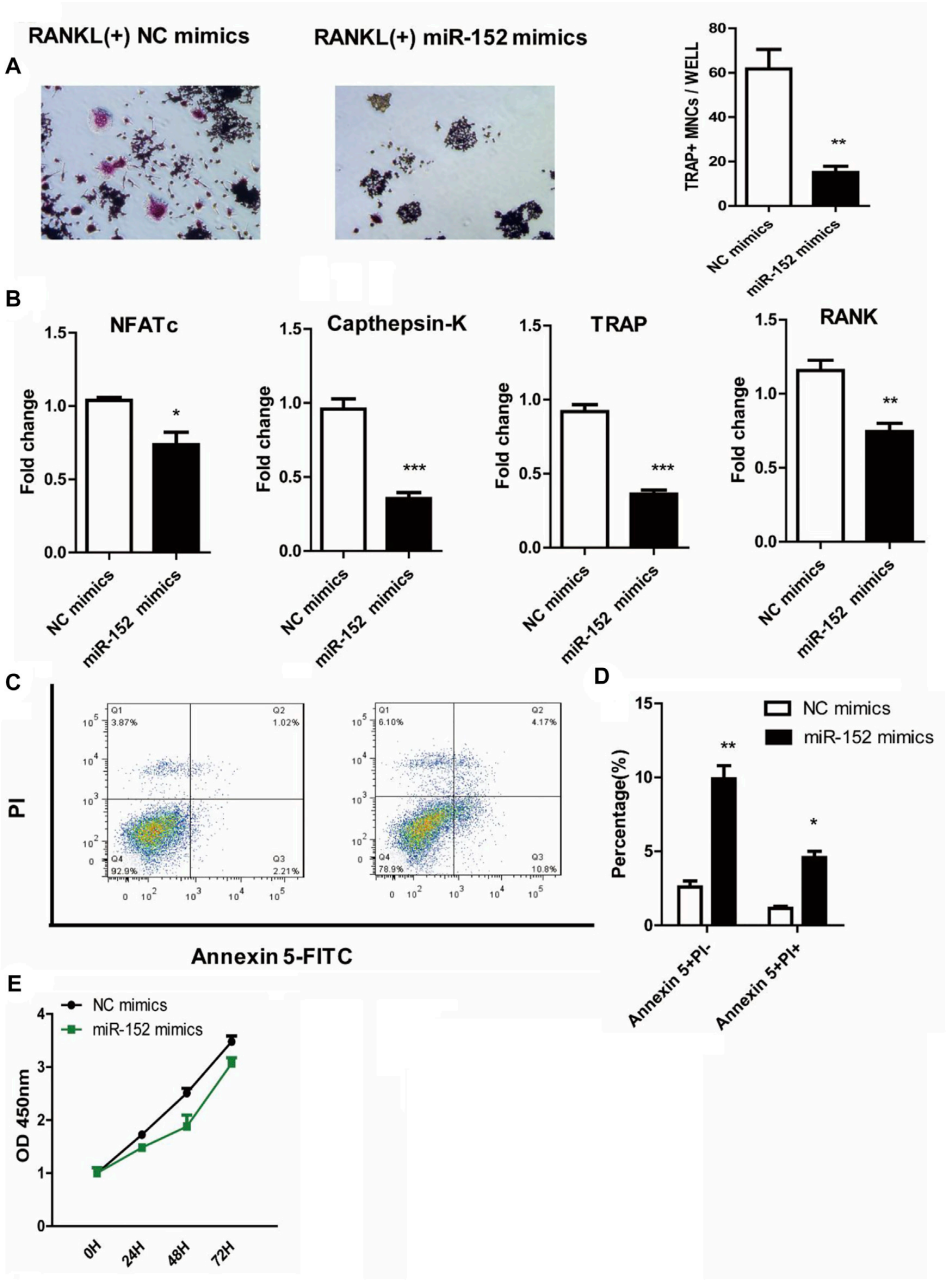
miR-152 inhibits the RANKL-induced osteoclast differentiation in RAW 264.7 cells **(A)** Representative images and quantification of RANKL-induced osteoclasts transfected with NC mimics or miR-152 mimics were analyzed for TRAP + cells, Magnification: 100* **(B)** RT-qPCR analysis of osteoclast differentiation-related genes (NFATc1, Cathepsin K, TRAP, and RANK) expression in mature osteoclasts transfected with NC mimics or miR-152 mimics **(C)** After staining with Annexin 5-FITC and propidium iodide (PI), followed by analysis using a flow cytometer. Early apoptotic cells were Annexin5^+^ and PI^−^ and late apoptotic cells were Annexin5^+^ and PI^+^ **(D)** Analysis of mature osteoclasts apoptosis transfected with NC mimics or miR-152 mimics **(E)** RAW264.7 cells were cultured with M-CSF in the absence or presence of miR-152 mimics siRNA for 3 days, the proliferation ability of early osteoclast precursors was analyzed by CCK-8 assay. Bars represent the mean ± SEM.*p < 0.05, **p < 0.01, ***p < 0.001. The data are representative of three independent experiments.

We used starbase (http://starbase.sysu.edu.cn/) to identify the potential binding sites between HOTAIR transcript and miRNAs, such as miR-6807-3p, miR-148a/b-3p, miR-1227-5p, and miR-152 (Figure 4A). Among them, miR-152 expression in the craniosynostosis children’s group was significantly upregulated (Figure 4B) compared with that in PBMCs of healthy controls. Moreover, the levels of miR-152 were notably increased in RAW264.7 cells treated with HOTAIR siRNA compared with those in cells treated with negative control siRNA (Figure 4C). The RIP results showed that HOTAIR was observed in miR-152-overexpressing RAW264.7 cells (Figure 4D), verifying the interaction between HOTAIR and miR-152. The TRAP staining results indicated that miR-152 overexpression could remarkably reduce the number of TRAP-positive multinucleated osteoclasts (Figure 5A). The mRNA levels of osteoclast differentiation-related genes, including *NFATc1, Cathepsin K, TRAP*, and *RANK* were significantly downregulated in miR-152 mimic-treated group (Figure 5B). Meanwhile, miR-152 mimic administration enhanced osteoclast apoptosis (Figure 5C,D). At the same time, it had little effect on precursor proliferation (Figure 5E), similar to the knockdown of HOTAIR data.

### HOTAIR-miR-152-CAMKIIαaxis Regulates Osteoclast Differentiation

**FIGURE 6:**
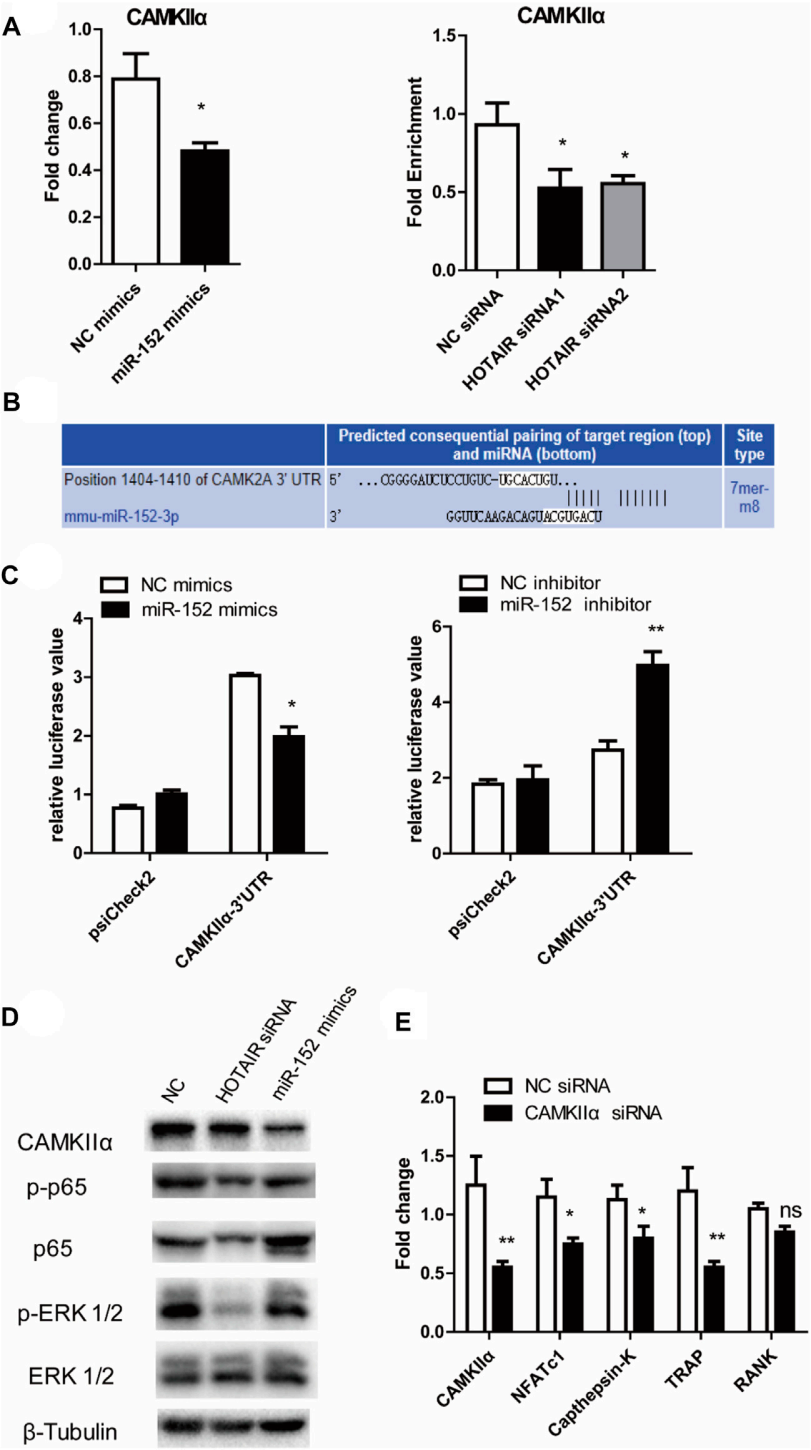
HOTAIR-miR-152-CAMKIIα axis regulated osteoclast differentiation and apoptosis through NF-κB and MAPK/ERK 1/2 pathway **(A)** Expression of CAMKIIα mRNA in both miR-152 overexpressed (left) and HOTAIR-knockdown osteoclasts (right) was downregulated **(B)** Predicted binding sites for miR-152 in the 3′-UTR of CAMKIIα **(C)** RAW264.7 cells were transfected with the control construct (psiCHECK-2), or a construct encoding the wild-type CAMKIIα 3′-UTR, in addition to the miR-152 mimics (left) or miR-152 inhibitor (right). After 24 hs, luciferase activity in RAW264.7 lysates was detected **(D)** After transfection with HOTAIR siRNA or miR-152 mimics, the protein levels of CAMKIIα, Phosphorylated p65, p65, Phosphorylated ERK 1/2, and ERK 1/2 in mature osteoclasts were examined by western blotting. β-Tubulin was used to confirm equal protein loading **(E)** Effect of CAMKIIα on osteoclast differentiation-related gene expression in CAMKIIα siRNA-treated osteoclasts. Bars represent the mean ± SEM. *p < 0.05, **p < 0.01. The data are representative of three independent experiments.

qRT-PCR results showed that miR-152 overexpression or HOTAIR silencing could significantly reduce the mRNA levels of CAMKIIα in RAW264.7cells (Figure 6A). The luciferase assay revealed that miR-152 regulated the luciferase activity in a CAMKIIα 3′UTR-dependent manner (Figure 6B,C). Moreover, the results showed that HOTAIR knockdown or miR-152 overexpression could strongly attenuate p65 and MAPK/ERK 1/2 phosphorylation and protein level of CAMKIIα (Figure 6D). Furthermore, the mRNA expression levels of osteoclast differentiation-related genes *NFATc1, Cathepsin K, TRAP,* and *RANK* were significantly downregulated in the absence of CAMKIIα (Figure 6E).

## Discussion

In this study, our data showed that HOTAIR expression was significantly downregulated in PBMCs from children with craniosynostosis. However, approximately 50% of normal individuals had PBMC HOTAIR expression equal to or lower than patients with craniosynostosis, suggesting that low HOTAIR expression in PBMCs is related to craniosynostosis but might not be a suitable biomarker for craniosynostosis. The results of cell proliferation and apoptosis assays indicated that silencing of HOTAIR could inhibit osteoclast differentiation and increase cell apoptosis. Moreover, the luciferase reporter assay results showed that the regulatory axis, HOTAIR-miR-152-CAMKIIα, was the regulatory mechanism of HOTAIR in the osteoclast function and development of craniosynostosis. All those results implied that HOTAIR might regulate osteoclast formation to participate in the development of craniosynostosis.

There is increasing evidence that miRNAs are involved in the regulation of osteoclast differentiation and bone resorption (Tang et al., 2014). Ma et al. have shown that inhibition of miR-152-3p impairs osteoclastogenesis *in vitro* and reduces the osteolytic lesions while preserving trabecular architecture *in vivo*, suggesting that miR-152-3p promotes osteoclastogenesis (Ma et al., 2021). On the contrary, Xu et al. have found that overexpression of miR-152 alleviates bone disruption in an intrabone multiple myeloma mouse model by targeting Dickkopf-1 (Xu et al., 2015). Since inhibition of Dickkopf-1 has been shown to increase osteoblastic differentiation while reducing osteoclast activity in a myelomatous mouse model (Yaccoby et al., 2007), the findings of Xu et al. suggest that miR-152 may suppress osteoclastogenesis. Consistently, our data indicate that miR-152 inhibits osteoclast formation and suggest that HOTAIR may regulate osteoclast formation by interfering with miR-152 expression and function.

The past decade has elucidated many important mechanisms in the biology of skull suture, including osteoblast dysfunction and regional dura involvement in regulating suture fusion (Bradley et al., 1997; Levine et al., 1998). These studies have become the basis for the development of molecular therapy. However, bone biology depends on the interaction between osteoblasts and osteoclasts. Therefore, bone pathology may also involve 2 cell types. Indeed, HOTAIR also affects osteoblastogenesis. Yuan et al. have shown that overexpression of HOTAIR inhibits osteogenic differentiation while promoting adipogenic differentiation of bone marrow stromal cells (Yuan et al., 2020). Wei et al. have found that HOTAIR inhibits osteogenic differentiation by suppressing miR-17-5p expression (Wei et al., 2017). HOTAIR also inhibits osteoblast differentiation of rat bone marrow stromal cells (Shen et al., 2019). Therefore, the results observed in our animal model and the possible role of HOTAIR in craniosynostosis seem to be related to the fact that HOTAIR favors bone resorption while inhibiting bone formation.

CaMKIIα is a vital gate that could control the structural, functional, and behavioral expression of synaptic memory. This may result in brain hypoplasia and craniofacial anomalies, including craniosynostosis and other anomalies (Yamagata et al., 2009). However, the rationality of the HOTAIR-miR-152-CAMKIIα pathway in the progression of craniosynostosis has not been explored in previous studies cordially. Nevertheless, the data of this study suggest that the HOTAIR-miR-152-CAMKIIα regulatory axis may be involved in forming osteoclasts during the occurrence and development of cranial suture injury. CAMKIIα is a serine/threonine protein kinase, which could play an important role in mediating NF-κB and MAPK/JNK signal transduction (Chang et al., 2008). NF-κB and MAPK signaling pathways play an essential role in regulating RANKL induced osteoclast formation. In the present study, CAMKIIα was identified as a direct target of miR-152. We found that HOTAIR knockout or miR-152 overexpression could inhibit CAMKIIα expression in RAW264.7 cells and weaken the activation of NF-κB. These data suggest that the HOTAIR-miR-152-CAMKIIα regulatory axis may be involved in forming osteoclasts during the occurrence and development of skull suture injury. However, the miR-152/CAMKIIα axis in osteoclast function and the occurrence and development of cranial suture disease are still unclear. It has been reported that miR-152/CAMKIIα axis is involved in immune homeostasis and immune regulation. Their data showed that miR-152 inhibited cytokine production, including IL-12, IL-6, TNF-α, and IFN-β. This has been upregulated MHC class II expression and inhibited DC-initiated Ag-specific T cell proliferation by targeting CAMKIIα(Liu et al., 2010). Therefore, it is suggested that miR-152/CaMKIIα may play an important role in osteoclast function. Through immunomodulation, it can cause the occurrence and development of craniosynostosis. This needs to be further explored.

In conclusion, craniosynostosis is the second most common cranial facial anomaly. The premature fusion of cranial sutures leads to deforming the skull shape and restricts brain growth. In this study, our findings demonstrated the role of HOTAIR in craniosynostosis through modulating miR-152 and its target gene CAMKIIα. This study provided a novel insight for understanding the potential molecular mechanism of osteogenic differentiation in craniosynostosis.

## Impact Statement

Craniosynostosis is a common congenital craniomaxillofacial malformation, and its pathogenesis is still unclear at present. Most studies have focused on the effect of osteoblasts on cranial suture. This study explores the opening and closing of cranial suture from the perspective of osteoclasts, and the role of lncRNA HOTAIR on the biological activity of osteoclasts, so that to explore a new idea on the mechanism of craniosynostosis.

## Data Availability

The raw data supporting the conclusions of this article will be made available by the authors, without undue reservation.
